# New Bioactive Calcium Silicate Cement Mineral Trioxide Aggregate Repair High Plasticity (MTA HP)—A Systematic Review

**DOI:** 10.3390/ma14164573

**Published:** 2021-08-14

**Authors:** Mirona Palczewska-Komsa, Kinga Kaczor-Wiankowska, Alicja Nowicka

**Affiliations:** Department of Conservative Dentistry and Endodontics, Pomeranian Medical University in Szczecin, Powstancow Wielkopolskich 72, 70-111 Szczecin, Poland; mpalczewskakomsa@op.pl (M.P.-K.); nowicka6@gmail.com (A.N.)

**Keywords:** mineral trioxide aggregate, MTA repair HP, bioactive calcium-silicate cement, review

## Abstract

Bioactive calcium silicate cement Mineral Trioxide Aggregate (MTA) has been used for years as a gold standard in intravital pulp treatment and specialist endodontic procedures. Owing to flaws of the material, the manufacturers have been trying to enhance and produce materials showing improved physical, chemical and biological parameters. One of the new calcium-silicate cements based on mineral trioxide aggregate, however without some flaws exhibited by the cement, is Mineral Trioxide Aggregate Repair High Plasticity (MTA HP). The aim of the present paper was a systematic literature review concerning the MTA HP material used nowadays in dentistry, as a review of its specific features. The present paper is the first article providing a systematic literature review on MTA HP. The aim of the present article is the better understanding of MTA HP properties, which can aid the decision-making process in endodontic treatment.

## 1. Introduction

Bioactive materials have been successfully used in dentistry, particularly in endodontic procedures. The bioceramics applied in the treatment are classified as bioinert, bioactive and biodegradable materials [1,2,3,4]. The shared feature of the ceramic materials is their role in minimally invasive endodontics and regenerative endodontics. Moreover, these materials can be used for root canal sealing and root cavity filling [1].

The leading bioceramic material that has been used as a gold standard in biological treatment of the pulp and specialist endodontic procedures is Mineral Trioxide Aggregate MTA [5,6]. MTA is available in two variations, depending on the color, i.e., gray and white MTA. The major difference between gray MTA (ProRoot MTA, Densply Sirona) and white MTA (White ProRoot MTA, Densply Sirona) is the concentrations of Al_2_O_3_, MgO and FeO [7,8]. The traditional MTA compositions have some drawbacks: discoloration of the tooth and marginal gums, long setting time, and difficult handling [6,9]. The difficult handling of MTA seems to be aggravated in such procedures as root, furcation perforation or filling of root-end cavities. [5,10]. Recently, new formulations have been introduced [6]. Among them, MTA HP, (Angelus, Londrina, PR, Brazil) has been proposed. According to the manufacturer, it is an endodontic restorative cement of high-plasticity consisting of mineral oxides in the form of fine hydrophilic particles. The formula of MTA HP maintains all the chemical and biological characteristics of the original MTA preparation. Table 1 shows the composition of MTA (ProRoot MTA, Densply Sirona) cement used as a gold standard in endodontics and the properties of MTA HP material under study [11,12,13].

MTA HP is indicated for the following: iatrogenic and carious furcation the treatment of root canal perforation; periapical surgery with retrograde root canal filling; the root canal treatment of root perforation due to internal resorption; pulp capping; pulpotomy (the removal of the affected pulp from the crown of the tooth while maintaining viability and function of the pulp) [14]. The present paper is the first to provide a systematic literature review concerning MTA HP material. The aim of the study was to analyze the physicochemical and biological properties in in vitro and in vivo research with the use of MTA HP material. This will improve better understanding of MTA HP properties which can aid the decision-making process in endodontic treatment.

## 2. Material and Methods

### 2.1. Review Questions 

**Figure 1:**
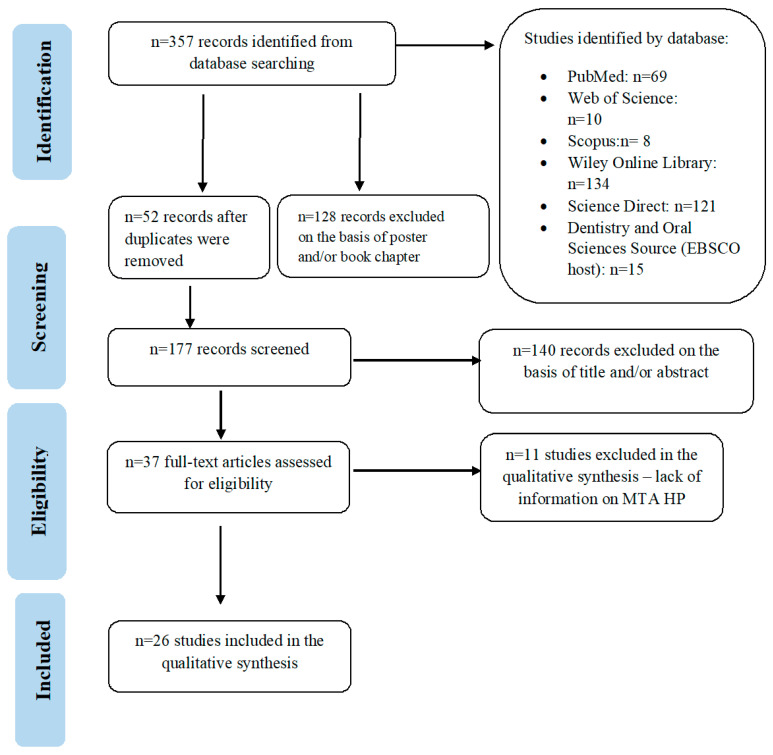
Search flow diagram compliant with PRISMA guidelines.

The review of the literature was based on the Preferred Reporting Items for Systematic Reviews (PRISMA) (Figure 1) [15]. This article aims to answer the following questions:

1. Which biological, physical and chemical parameters of MTA HP influence its therapeutic efficacy?

2. What are the disadvantages and advantages of MTA HP in comparison with other calcium silicate cements?

### 2.2. Information Sources and Search Strategy

Five databases (PubMed/MEDLINE, Web of Science, Scopus, Dentistry and Oral Sciences Source (EBSCOhost, Wiley Online Library and Science Direct) were searched electronically by two independent reviewers (M.PK and K.KW) for publications involving information about MTA HP. Any disagreements were resolved by mutual discussion and consensus with an experienced researcher (A.N.). Publications were searched without a year limit. The last search was conducted on 22 May 2021. The search phrases are presented in Table 2. All papers were imported into the Mendeley (2020 Mendeley Ltd., Elsevier) program for the management of scientific bibliography. Having removed the duplicates, all articles (titles, abstracts) were examined. Articles were extracted based on the inclusion criteria listed below:

1. In vitro and in vivo studies that evaluated MTA HP.

2. Publications concerning the physicochemical and biological properties of the MTA HP. 

### 2.3. Eligibility Criteria

The research included in vitro and in vivo studies that compared the clinical effectiveness of MTA HP with different bioceramic materials. The author excluded articles of which the full texts were not available in English. All exclusion and inclusion criteria are described in Table 3. Data concerning the papers, properties, application material, the number of cases/controls, the type of clinician problem, observation time, retention, marginal adaptation and solubility were extracted from the selected papers by means of a standardized sheet in Microsoft Office Word 2010 (Microsoft Corporation, Redmond, WA, USA).

## 3. Results

### 3.1. Quality Assessment

The quality of in vitro and in vivo studies was assessed using a modified methodological index for non-randomized studies (MINORS) modified by the authors [16]. In the MINORS scale, the following were taken into account: clear aim, clear application protocol of HP MTA, inclusion of additional patients or animals, prospective data collection, justification of the sample size, observation period, endpoints relevant to the study purpose, blind analysis, adequate control group, contemporary groups, baseline group equivalence and adequate statistical analyzes. The items were rated: 0, not reported; 1, introduced but inadequate; and 2, introduced and appropriate. All twelve items were used to evaluate the in vitro studies, while the first eight items were assessed for in vivo studies. The ideal result for comparative studies is 24, and for non-comparative studies 16 [16]. Test quality classifications were rated as: poor (0–5), good (6–10) and good (11–16) for in vivo tests, and poor (0–8), good (9–16) and good (17–24) for in vitro studies [17]. The results for each item, the overall result and the quality of the study are presented in Table 4 and Table 5 for the in vivo and in vitro studies, respectively.

### 3.2. Description of the Results

The database search produced 357 records in total. However, it must be emphasized that not all databases allowed the application of all the inclusion and exclusion criteria, therefore, at a subsequent phase of selection, the research papers not meeting the criteria were excluded by hand and the duplicates were eliminated. Additionally, owing to lack of information by the manufacturer concerning the time since when the product has been commercially available, the database search was not narrowed to particular time period. The results were thoroughly selected with respect to inclusion criteria and 26 research papers were eventually qualified to the analysis. Next, the research papers were divided according to the discussed parameters of MTA HP.

A total of 16 papers were included into the group discussing the physicochemical parameters of MTA HP [5,16,17,23,29,30,31,32,33,34,35,36,37,38,39]. The analysed selected physicochemical parameters of MTA HP, together with the most important results are given in Table 6. 

The group of research papers on the biological properties comprised a total of 18 scientific articles, including: seven studies on human cells in vitro [12,23,24,25,26,27,28], seven on Wistar rats in vivo [6,9,18,19,20,21,22], two research papers on mice in vitro [21,29] and three papers cells in vitro [37,38,39]. A brief description of the aforementioned studies is presented in Table 7. However, it should be noted that for the purpose of clarity, the table presents only the maximum time of the in vitro and in vivo studies. It should be emphasized that some authors discussed both the physical as well as biological properties in a single research paper, or discussed the biological properties in two species, which can mistakenly suggest that there were more than 26 papers under analysis. For ease of reference, Table 8 summarizes the research studies on the MTA HP material.

## 4. Discussion

MTA-based materials are classified as bioceramic materials. Generally, bioceramic materials are biocompatible ceramic compounds originating both in situ as well as in vivo from various chemical processes. They show superior biocompatibility due to biological characteristics resembling those of hydroxyapatite. During hydration, bioceramics generate numerous compounds, e.g., hydroxyapatite, which show the ability to induce regenerative reaction in the human organism. At contact with the bone, mineral hydroxyapatite shows osteoconductive properties which results in the formation of a new bone in the interphase. Additionally, bioceramic materials exhibit inherent osteoinductive ability due to the reported ability to absorb osteoinductive substances near the bone healing site [1,2]. Bioceramic materials are biocompatible and have antibacterial properties. The latter is due to in situ precipitation of the material, following the setting time, which results in bacteria sequestration. The materials take the form of porous preparation with nanocrystals of 1–3 nm in diameter, thus preventing bacteria adhesion. At times, fluoride ions are the constituents of apatite crystal, and the resulting nanomaterial shows antibacterial properties. Furthermore, bioceramics can be used jointly with synthetic hydroxyapatite [1,4]. The MTA HP cement was developed to overcome the disadvantages of previous MTA materials. In particular, the materials used for retrofilling the canals require greater plasticity, shorter setting time and easy insertion into the root [12]. The discussion describes the selection of the most important physicochemical and biological parameters of the MTA HP as a bioceramic material. Whenever possible, the properties of the HP MTA material were compared to other MTA materials. The most significant physicochemical parameters with respect to results obtained by different authors are presented in Table 6, Table 7 and Table 8.

### 4.1. Physicochemical Properties

#### 4.1.1. Setting Time

The setting time of endodontic cement should be less than 10% of the time specified by the manufacturer. The classic calcium-silicate materials dissolve in tissues after some time following the application, which is an undesirable feature of these materials. However, unlike calcium hydroxide, MTA HP shows very low solubility [13]. According to Galarça et al. in 2018 [29], the MTA repair HP cement had a longer setting time than MTA Angelus. In the MTA HP material, calcium tungstate was used as a radiation sedative instead of bismuth oxide [29]. The use of calcium tungsten is beneficial because it has a stimulating effect on the physicochemical, antibacterial and biological properties of MTA cement [40]. Additionally, in the case of MTA HP, it is assumed that the plasticizer included in the liquid formulation of this cement favored the reduction of the setting time [29]. In turn, Jiménez-Sánchez (2019) stated that the short setting time measured for MTA HP is correlated with the precursor powder high surface area, the absence of compositional sulphate phases, and high Al content. Calcium sulphates affect the rate of chemical bonding. The less sulphate, the shorter the setting time of the MTA HP material [36]. This is contrary to the findings of Guimarães et al., 2018, which showed that MTA HP showed similar end-setting time values as MTA Angelus, but that the presence of a plasticizer could increase solubility and porosity [30]. However, Acris De Carvalho et al., 2021, indicated that when MTA HP was activated by ultrasound, the setting time was reduced when compared to the control group of MTA HP without ultrasound activation. The authors provide the following explanation: when ultrasonic activation is used, there is an air vortex and changes in hydrostatic pressure, which favors the formation of cavitation, causing an increase in the temperature of the material. This increases the hydration and reaction kinetics [32,41]. The presence of water in the cement leads to the transformation of calcium oxide into calcium hydroxide and, additionally, shortens the setting time [42].

#### 4.1.2. Flow and Dimensional Change

Endodontic cements cannot have a diameter smaller than 20 mm in the flow test [43]. According to the study by Acris De Carvalho et al. (2021) MTA HP met the standard only following activation with ultrasound [32]. Chemical transformations occurring as a result of the material’s contact with water in the process of cement hydration and an increase in temperature may affect the decrease in cement particle diameter and the reduction of the viscosity of the cements, favoring the flow [44]. In turn, as pointed out by Bodanezi et al. in 2008, the changes in the particle size contribute to a decrease in sealing ability of the cement and may favor the development of infection, thus leading to treatment failure [45]. 

#### 4.1.3. Solubility and Water Sorption

As for solubility, testing observed that it should not exceed 3% of the material’s mass [43]. In the case of MTA HP, following the preparation of the material according to the manufacturer’s instructions, there is an increase in mass due to water absorption. The pores form on the surface as well as inside the material. These empty spaces weaken the structure of the cement and are susceptible to the activity of the solvent which allows water absorption [46]. The conventional material of the MTA was compared with that of the MTA HP. It was found that conventional MTA showed the lowest values for solubility, open pore volume, apparent porosity and water sorption in comparison to MTA HP [30]. This difference between the conventional MTA and MTA HP, both of which have similar compositions, could be due to the plasticizer contained in the mixing liquid of MTA HP [30] Hydration contributes to increasing the mass of cement, loosening its structure and creating voids [29,47]. Ultrasonic activation reduces the occurrence of this unfavorable phenomenon [32].

#### 4.1.4. Film Thickness, Bond and Compressive Strength

With respect to film thickness, Galarça et al. (2018) stated that MTA HP had a significantly lower film thickness (but it still did not meet the ISO 6876 standard) than MTA Angelus. This property may be explained by observations that are in agreement regarding a finer particle size. Moreover, it was also found that MTA HP compared to conventional MTA and Biodentine shows lower bond strength and marginal adaptation that can be enhanced by ultrasound [31]. The particle size of the new cement was slightly smaller than that of MTA Angelus [29]. However, a study by Silva et al. in 2016 showed a new methodological aspect. Pores were made in the dentin fragments and care was taken to ensure that the MTA HP material and time points were further compared using the same dentin sample [5,48]. The authors concluded that HP MTA showed better bond strength and resistance to thrust compared to white MTA but worse than Biodentine. Replacing bismuth oxide with calcium tungstate as a radiopacificator in MTA HP could explain the better performance of this cement compared to white MTA [5]. Calcium tungstate contributes to higher calcium release, promoting higher biomineralization [49]. Regarding compressive strength, when compared with the conventional MTA, lower values were recorded for MTA HP following 24 h, and the values showed an increase with time and were higher than that of the conventional MTA following 28 days. The improvement of compressive strength in time may reduce susceptibility to fracturing and cracking of the cement. This is a desired feature as it makes the material more durable to occlusal stress [29].

#### 4.1.5. Marginal Adaptation and Microleakage

It was found that the acoustic force induced by ultrasounds has a positive effect on the adaptation of the material to the walls of root canals. Therefore, better adaptation of the MTA HP material increases the adhesion [30].

According to the results by Aguiar et al., 2019, MTA HP maintains the color stability and does not result in tissue discolouration owing to lack of bismuth oxide. This feature is an undisputed advantage over the conventional MTA containing bismuth oxide as a radiopacifier [31]. Metlerska et al. (2021) analyzed the effects of 10% and 40% citric acid (CA) on the color of calcium silicate-based cements (CSCs) in comparison to the effects of common root canal irrigants [35]. It was found that MTA repair HP was least sensitive to NaOCl solution of all the tested CSCs materials. It also exhibited the slightest spectrophotometric color changes upon immersion in CHX and EDTA. Only in contact with CA solution did its surface structure disintegrate. Irrigation solutions do not cause darkening of MTA HP, which could be visible to the naked eye [35]. 

#### 4.1.6. Main Compositional Phases and pH

The main components of MTA HP are: CaWO_4_, Ca_3_SiO_5_ and Ca_2_SiO_4_ as the main phases of the composition. The content of calcium aluminate improves the biological response of MTA HP [27,36]. In several scientific studies, the chemical composition of HP MTA was determined using energy dispersion X-rays [14,15,17,36]. Jiménez-Sánchez et al. (2020) found that the structure of the MTA HP tricalcium silicate particles ensures a very close contact between the calcium silicate and calcium aluminate and thus favors the hydration reaction. In this way, hydrated calcium aluminate silicate is formed which is responsible for the biocompatibility and speed of the reaction [38]. El Reash et al. (2019) found that MTA HP was less alkaline (pH = 11.5) compared to iRoot BP Plus (pH 12.1) [27].

#### 4.1.7. The Release of Calcium Ions

An important aspect of endodontic treatment is the proper formation of mineralized tissue. Biomineralization is the synthesis of a mineral by a living organism. In turn, bioactivity is the deposition of calcium phosphate deposits on the surface of materials in the buffer. The buffer contains ions close to human plasma [50,51,52]. The most important thing in the pulp capping is the release of calcium ions. Calcium influences the differentiation of pulp cells and thus the mineralization of hard tissue [52,53]. MTA HP releases calcium most intensively in the first month after application, compared to conventional MTA, which showed the highest release of calcium after 1 week [30]. The release of calcium ions takes place by dissociation of calcium hydroxide, the by-product of which is the hydration of Ca_2_ + and OH– ions, tricalcium and dicalcium [54].

The growth of calcium phosphate apatite crystals is the environment for the differentiation and colonization of stem cells and osteoblasts [5]. Scanning electron microscopy analysis provided qualitative and semi-quantitative measurements of calcium and atomic phosphorus. The high intensity of the Ca and P peaks detected in the analysis indicated the precipitation of amorphous sediments corresponding to calcium phosphate [55]. The Ca:P atomic ratio one month after application was lower for MTA HP material compared to conventional MTA. This result may be correlated with the presence of a plasticizer [30]. On the other hand, the MTA HP material is more effective regarding the bioactive reaction rate compared to the Pro and Neo materials [30]. This is in line with the studies by Jiménez-Sánchez et al., who conducted an in vitro bioactivity assessment of MTA HP material. The authors believe that MTA HP can be used as an endodontic repair cement due to its high biocompatibility [39].

### 4.2. Biological Properties

#### 4.2.1. Inflammatory Response

Out of the biological parameters, the inflammatory response of the tissues in contact with MTA HP is discussed particularly often [6,9,12,18,20,21]. From the clinical perspective, the most desirable situation is that of a lack of such a response, which would indicate an ideal biocompatibility of the material covering the tissue. However, the research papers under analysis show that MTA HP causes a local inflammatory reaction in contact with living tissue. The in vivo studies conducted on rats showed that MTA HP was implanted in the dorsal region for some time. Next, having euthanized the animals, the tissue with an implanted MTA HP was assessed in terms of inflammatory reaction [6,9,18,20,21]. The most severe inflammatory reaction was found in the first few days following the contact of the tissue with MTA HP and showed a decrease in time. After 30 days, the inflammatory response was found to be slight [6,9,20,21]. However, none of the research articles under study provides an assessment of the final resolution of the inflammatory response. The longest experiment (90 days) was conducted by Benetti et al. in 2018. Yet, even after such a long time, the authors still recorded the presence of single inflammatory cells [20]. An interesting observation was made by Cosme-Silva et al., 2019, who observed an increase in the tissue inflammatory infiltrate at contact with MTA HP in rats with spontaneous hypertension as compared to rats with normal blood pressure [18]. The authors note that the host’s response to substances released by the materials is a major factor influencing the progression and type of inflammatory infiltrate. Immune cells release cytokines and growth factors that play a key role in the inflammatory process. However, the inflammatory response of tissues in contact with cement may be exacerbated in hypertension. The reason for this is an increase in lymphocyte infiltration and multiplication, and an increased concentration of pro-inflammatory cytokines: interleukin-1 beta (IL-1β), interleukin-6 (IL-6) and tumor necrosis factor alpha (TNF-α) [18]. Barczak et al., 2021 investigated the MTA HP formula with respect to its effect on the inflammation process involving the tooth and periodontal tissues. The experiments were conducted in vitro on human monocytes of THP-1 cell line and macrophages obtained from THP-1 cells. It was found that MTA HP showed no activation of THP-1 monocytes and did not alter MMP-2 and MMP-9 protein expression in the cultured monocytes/macrophages. The crucial regulatory role of MMPs in terms of cell differentiation and migration processes, growth factors, angiogenesis, as well as development of inflammation has been confirmed [12]. Additionally, the inflammatory reaction caused by MTA HP was compared to that of other bioceramic materials. In the study by Benetti et al., 2019, it was found that in the group of rats with MTA HP and white MTA-Angelus implanted into the subcutaneous tissue, most of the samples had a mild inflammatory infiltrate [21]. However, in the group of rats with an implanted Bio-C Repair, there was only a mild to absent inflammatory reaction. Delfino et al., 2021 [19], assessed the inflammatory response elicited by Bio-C Pulpo, MTA HP and white MTA [19]. The authors concluded that the materials did not promote an inflammatory response and that the materials were biocompatible [19]. However, it must be observed that none of the aforementioned research papers on inflammatory response to MTA HP provides the time after which the inflammatory reaction recedes following the contact with MTA HP.

#### 4.2.2. Cytotoxicity

Another parameter discussed in the literature on the subject and analysed in the present paper is the cytotoxicity of MTA HP. This parameter is determined in most publication with MTT (3-[4,5-dimethylthiazol-2-yl]-2,5 diphenyl tetrazolium bromide) assay [56]. Ferreira et al. (2019) and Benetti et al. (2019) found that in vitro, cell viability depends on the degree of material dilution. The less the material is diluted, the less viable are the cells [6,21]. It is worth mentioning that the results are similar with respect to mice cells (fibroblast-like cells) as well as human cells (NHOst human bone primary osteoblasts) [6,21]. El Reash et al., 2021, investigated the effects of MTA HP on the proliferation of human Dental Pulp Stem Cells (hDPSCs) and found a significant increase in hDPSCs cellular proliferation as compared to control group (culture complete medium without MTA HP) [29]. This may be due to the leaching of calcium ions [28]. The released calcium is conducive to apatite formation: the tricalcium silicate- based cements, when in contact with interstitial fluid, released calcium, silicate hydrate and calcium hydroxide [57]. Calcium hydroxide releases calcium ions necessary for cells adhesion, migration, differentiation and proliferation of cells. Moreover, calcium plays a key role in fibroblast adhesion [58,59]. It was proved that more Ca ions are released from MTA HP at pH 5.2 than at pH 7.4 [26]. The explanation of the relationship is not clear due to insufficient number of analyses of MTA HP in contact with an acidic environment. Additionally, Tomás-Catalá at al. studied evaluate the in vitro cytotoxicity of MTA HP, Neo MTA Plus, and Biodentine, on human dental pulp stem cells (hDPSCs) [24]. The cytotoxicity of MTA HP, Neo MTA Plus and Biodentine materials was compared. It was found that all tested materials showed a normal level of cytocompatibility with hDPSC cells and good cell migration rates, although the best parameters were obtained for Biodentine [24].

#### 4.2.3. Apoptosis/Necrosis

Several authors are in line with respect to low ability of MTA HP to generate apoptosis of cells in contact with the material. The estimated cell viability following the contact with MTA HP is more than 90% [6,9,26].

#### 4.2.4. Cellular Adhesion 

The MTT test is an adequate method of quantitative assessment of cell viability/proliferation [26]. However, it only provides an investigation of a single aspect of biocompatibility and is not to be used alone to determine if the material is cytocompatible or not [60]. Therefore, apart from MTT test, the ability of the material to provide an adequate surface for cellular adhesion is analyzed with the scanning electron micrographs (SEM), the extracellular calcium deposition (bioactivity) is quantitatively assessed with Alizarin Red staining to fully understand the properties of the materials. Research shows that after 3 days of cultivation, hPDLSCs attached to the MTA HP material when exposed to various environments. No differences were observed between the material and the acidic or neutral environment [26]. Additionally, after 72 h, Ferreira et al., 2019 observed a large number of cells covering the disc surface coated with MTA HP and cultured cells [6]. However, as has been demonstrated by El Reash et al., 2021, after 7 days, the cells (human hDPSCs) exhibited characteristics of fibroblastic morphology, reflecting good attachment to the MTA HP [28]. On the other hand, however, it must be observed that bioceramic materials show different adhesion to tissues, as has been demonstrated by [23]. The authors compared the properties of MTA HP, Neo MTA and Biodentine on hDPSCs cells obtained from impacted third molars. They found that MTA HP causes a significantly lower cell adhesion to the material when compared to Neo MTA and Biodentine [23]. The authors attribute this to differences in chemical composition of the elements between the analyzed materials. The presence of Sr in eluates is 10-times greater in MTA HP and 100-times greater in Neo MTA Plus as compared with Biodentine [23]. The presence of Sr, Al and S affects the cytotoxicity (in Neo MTA Plus and MTA HP), whereas their low amounts or lack (in the case of Biodentine) is connected with a markedly increased biocompatibility of the material [23]. 

#### 4.2.5. Mineralization

In a test with the use of Alizarin Red staining it was found that after 24 h in human osteoblastic cells there were more mineralization noodles than in the control group (culture complete medium without MTA HP) [25]. A significant increase in the amounts of mineralizing nodules formed, when compared to control group (culture complete medium without MTA HP), was also observed following 7 days in dental pulp stem cells (hDPSCs) [28]. In vitro tests conducted on rat cells demonstrated that MTA HP showed a superior mineralization ability of close to 100% [25,27]. It must be noted that some factors, such as arterial hypertension, contributed to a decrease in mineralization ability of the tissues covered with MTA HP [18]. Macedo et al., 2020 investigated the response of the laser photobiomodulation after filling a bone defect with MTA HP in a rat. They found that the irradiated group presented a clear narrowing of the medullary spaces, suggesting greater bone compaction. The authors conclude that laser photobiomodulation, when associated with the use of material MTA HP, has been good for the bone repair process [22].

#### 4.2.6. Antibacterial Effect

In terms of the antibacterial effect, MTA HP was assessed against other materials such as ACTIVA or iRoot BP [9]. With respect to facultatively aerobic Gram + ve (*S. aureus, S. mutans, E. faecalis* and *E. faecium*), MTA HP showed a marked inhibitory effect on *S. aureus, S. mutans* and *E. faecium*, however the effect was found to be even stronger regarding iRoot BP. Similarly, IRoot BP showed the superior antibacterial effect against *E. faecalis*. In terms of *P. anaerobius, A. israelii* and *P. gingivalis*, again iRoot BP as well as MTA HP showed an inhibitory activity greater than that of ACTIVIA. However, the inhibitory effect of MTA HP on anaerobes was found to be lower than that of iRoot BP. Additionally, it must be emphasized that MTA HP shows no antibacterial properties against *A. israelii* and *P. gingivalis.* Finally, MTA HP demonstrated a superior inhibitory effect on *C. albicans* when compared to iRoot BP, whereas ACTIVIA showed no antifungal activity [9]. According to the results of other studies, it was found that MTA HP exhibits antimicrobial activity mainly due to its high pH. However, it turns out that the MTA HP material is not effective against anaerobic bacteria [27].

Bioactive calcium-silicate cements are being increasingly used with a widening application options in endodontics and regenerative endodontics. There are a number of research studies describing in detail the physical and chemical properties of MTA HP. It transpires that an improvement of some chemical and biological properties has a negative effect on the physical parameters of the said material. A small number of studies provides the analysis of MTA HP in vivo in human teeth in clinical settings. Most research studies published to date is based on in vitro studies on laboratory animals (mice and rats).

## 5. Conclusions

The analysis of the conducted studies allows for the gaining of greater understanding by doctors prior to using the preparation and, consequently, the obtainment of improved treatment outcomes in clinical settings. However, more long-term in vivo studies with a larger sample size and proper clinical settings are required to extend the knowledge and draw a definitive conclusion on MTA HP.

## Figures and Tables

**Table 1:** Detailed manufacturer data on ProRoot MTA and MTA repair HP.

Material	Producer	Composition	Properties
Pro Root MTA	Dentsply Sirona, Tulsa, USA	Powder: tricalcium silicate (CaO)_3_·SiO_2_ dicalcium silicate (CaO)_2_·SiO_2_ tricalcium aluminate (CaO)_3_·Al_2_O_3_ bismuth oxide Bi_2_O_3_ gypsum CaSO_4_·2H_2_O Liquid: distilled water H_2_O	The handling can be difficult, the initial setting time is long (78 min), the use in the visible crown area may lead to tooth discoloration, the lowest radiopacity of all MTA materials
MTA Repair HP	Angelus, Londrina, Brasil	Powder: Tricalcium Silicate 3CaOꞏSiO_2_, Dicalcium Silicate 2CaOꞏSiO_2_, Tricalcium Aluminate 3CaOꞏAl_2_O_3_, Calcium Oxide CaO, Calcium Tungstate CAWO_4_ Liquid: Water and Plasticizer	Material solidifies when kept in a wet environment after spatulation, the initial setting time is approximately 15 min; absence of dental discoloration due to the CaWO_4_ radiopacifier used. radiopacity: nearly matches that of gutta-percha. More radiopaque thanPro Root MTA;

**Table 2:** The search phrases.

Database	Filtres	Number of Phrase	Search Phrases
Medline (PubMed) (69)	Abstract Free full text Full text Clinical Trial Journal Article Randomized Controlled Trial	1	(teeth) OR (tooth) Filters: Abstract, Free full text, Full text, Clinical Trial, Journal Article, Randomized Controlled Trial
	-	2	(((((MTA HP[Title/Abstract]) OR (MTA high repair[Title/Abstract])) OR (MTA high plasticity[Title/Abstract])) OR (MTA repair HP[Title/Abstract]) AND ((ffrft[Filter]) AND (fha[Filter]) AND (clinicaltrial[Filter] OR journalarticle[Filter] OR randomizedcontrolledtrial[Filter]) AND (fft[Filter]) OR (Calcium Silicate-Based))))) Filters: Abstract, Free full text, Full text, Clinical Trial, Journal Article, Randomized Controlled Trial
	-	3	(solubility) OR (Ca2+ ions release)) OR (Bond strength)) OR (Film thickness)) OR (Adhesive)) OR (discoloration)) OR (Radiopacity)) OR (Setting time)) OR (Inflammatory response)) OR (Cytotoxicity)) OR (Apoptosis)) OR (Mineralization)) OR (Antimicrobal effect)) OR (physicochemical properties)) OR (chemical properties)) OR (biological properities)) OR (properities) Filters: Abstract, Free full text, Full text, Clinical Trial, Journal Article, Randomized Controlled Trial
	-	1 AND 2 AND 3	(teeth) OR (tooth) AND(MTA HP[Title/Abstract]) OR (MTA high repair[Title/Abstract]) OR (MTA high plasticity[Title/Abstract])) OR (MTA repair HP[Title/Abstract]) OR (Calcium Silicate-Based) AND (solubility) OR (Ca2+ ions release)) OR (Bond strength)) OR (Film thickness)) OR (Adhesive) OR (discoloration)) OR (Radiopacity)) OR (Setting time)) OR (Inflammatory response) OR (Cytotoxicity)) OR (Apoptosis)) OR (Mineralization)) OR (Antimicrobal effect) OR (physicochemical properties) OR (chemical properties)) OR (biological properties) OR (properties)
Web of Science (10)	Web of Science Categories: Dentistry Oral Surgery Medicine Document Types: Article	1	TOPIC: (teeth) OR TOPIC: (tooth)
-	-	2	(TS = (MTA HP OR MTA high plasticity OR MTA repair HP)
-	-	3	(TS = (solubility OR Ca2+ ions release OR Bond strength OR Film thickness OR Adhesive OR discoloration OR Radiopacity OR Setting time OR Inflammatory response OR Cytotoxicity OR Apoptosis OR Mineralization OR Antimicrobal effect OR physicochemical properties OR chemical properties OR biological properties OR properties)
-	-	1 AND 2 AND 3	(TOPIC: (teeth) OR TOPIC: (tooth) AND (TS = (MTA HP OR MTA high plasticity OR MTA repair HP) AND (TS = (solubility OR Ca2+ ions release OR Bond strength OR Film thickness OR Adhesive OR discoloration OR Radiopacity OR Setting time OR Inflammatory response OR Cytotoxicity OR Apoptosis OR Mineralization OR Antimicrobal effect OR physicochemical properties OR chemical properties OR biological properties OR properties)
Scopus (8]) Wiley Online Library (134)	Language: English Subjects: Dentistry Journals Subjects: Dentistry	1	teeth OR tooth
-	-	2	MTA HP OR MTA high plasticity OR MTA repair HP
-	-	3	solubility OR ca2+ AND ions AND release OR bond AND strength OR film AND thickness OR adhesive OR discoloration OR radiopacity OR setting AND time OR inflammatory AND response OR cytotoxicity OR apoptosis OR mineralization OR antimicrobial AND effect OR physicochemical AND properties OR chemical AND properties OR biological AND properties OR properties
-	-	1 AND 2 AND 3	(teeth OR tooth) AND (mta AND hp OR mta AND high AND plasticity OR mta AND repair AND hp) AND (solubility OR ca2+ AND ions AND release OR bond AND strength OR film AND thickness OR adhesive OR discoloration OR radiopacity OR setting AND time OR inflammatory AND response OR cytotoxicity OR apoptosis OR mineralization OR antimicrobial AND effect OR physicochemical AND properties OR chemical AND properties OR biological AND properties OR properties)
Science Direct (121) Dentistry and Oral Sciences Source (15)	Subject areas: Medicine and Dentistry Article type: Research articles Language: English	1	Teeth OR Tooth
-	-	2	MTA HP OR MTA high plasticity OR MTA repair HP
-	-	3	Properties
-	-	1 AND 2 AND 3	(Teeth OR Tooth) AND (properties) AND (MTA HP OR MTA high plasticity OR MTA repair HP)

**Table 3:** Exclusion and inclusion criteria.

Criteria	Included	Excluded
Full text	Available	Unavailable
Publication language	English	Other
Type of publication	Journal article	Books, documents
Type of research	Clinical trail	-
	Randomized controlled	
	Research articles	
	Review	
	Meta-analysis	
	Case Report	
Subject area	Dentistry and oral surgery medicine	Other
	Materials	
Publication stage	Final, In press	Other

**Table 4:** Risk of bias according to the MINORS scale in vivo studies (modified methodological index for nonrandomized studies).

Source	Ferreira et al., 2019 [6]	El Reash et al., 2019 [9]	Cosme-Silva et al., 2019 [18]	Delfino et al., 2021 [19]	Benetti et al., 2018 [20]	Benetti et al., 2019 [21]	Macedo et al., 2020 [22]
Clear aim	2	2	2	2	1	1	2
Clear MTA HP application protocol	2	2	2	2	2	2	2
Inclusion of consecutive patients, animals	2	2	2	2	2	2	2
Collection of data	2	2	2	2	2	2	2
Justification of sample size	0	0	0	0	0	0	0
Follow-up period appropriate to the aim of the study	2	2	2	2	1	1	2
Endpoints appropriate to the aim of the study	2	2	2	2	2	2	2
Blinded analysis	0	0	0	0	0	0	0
Study quality	good	good	good	good	fair	fair	good

Numbers coding: 2, introduced and adequate; 1, introduced but inadequate; 0, not reported.

**Table 5:** Risk of bias according to the MINORS scale in vitro studies (modified methodological index for nonrandomized studies).

Source	Barczak et al., 2021 [12]	Benetti et al., 2019 [21]	Tomás-Catalá et al., 2017 [23]	Tomás-Catalá et al., 2018 [24]	Queiroz et al., 2021 [25]	Collado-Gonzlez et al., 2016 [26]	El Reash et al., 2019 [27]	El Reash et al., 2021 [28]
Clear aim	2	1	2	2	2	2	1	2
Clear MTA HP application protocol	2	2	2	2	2	2	2	2
Inclusion of consecutive patients, animals	2	2	2	2	2	2	2	2
Collection of data	2	2	2	2	2	2	2	2
Justification of sample size	0	0	0	0	0	0	0	0
Follow-up period appropriate to the aim of the study	2	1	0	2	2	2	1	1
Endpoints appropriate to the aim of the study	2	2	2	2	2	2	2	2
Blinded analysis	0	0	0	0	0	0	0	0
An adequate control group	2	2	0	0	2	2	2	2
Contemporary groups	2	2	0	0	2	2	2	2
Baseline equivalence of groups	2	2	0	0	2	2	2	2
Adequate statistical analyses	2	2	2	2	2	2	2	2
Study quality	good	good	fair	fair	good	good	good	good

Numbers coding: 2, introduced and adequate; 1, introduced but inadequate; 0, not reported.

**Table 6:** Selected physicochemical parameters of the MTA HP.

Material Characteristics	Activation Ultrasonic	Time Period	Value (Mean± Standard Deviation)	Source
Setting time (min)	no	120 ± 10 s	12.20 ± 1.09	Acris De Carvalho et al., 2021 [32]
	yes	120 ± 10 s	10.00 ± 0.70	
	-	30 s intervals	13.0 ± 1.0	Galarça et al., 2018 [29]
	-	-	85 ± 2.64	Guimarães et al., 2018 [30]
Flow (mm)	no	180 ± 5 s	9.98 ± 0.18	Acris De Carvalho et al., 2021 [32]
	yes	180 ± 5 s	10.95 ± 0.14	
		10 min	18.15 ± 1.10	Galarça et al., 2018 [29]
Dimensional change (%)	no	30 days	1.72 ± 0.62	Acris De Carvalho et al., 2021 [32]
	yes	30 days	3.67 ± 1.97	
Solubility (%)	no	24 h	−3.66 ± 1.01	
	yes	24 h	−0.86 ± 0.89	
	-	24 h	−2.77 ± 1.18	Galarça et al., 2018 [29]
	-	-	8.18 ± 1.74	Guimarães et al., 2018 [30]
Water absorption (%)	-	24 h	16.32 ± 2.92	Galarça et al., 2018 [29]
	-	-	14.96 ± 0.95	Guimarães et al., 2018 [30]
Ca_2_ + ions release (mg/L)	no	-	28.76 ± 0.93	Acris De Carvalho et al., 2021 [32]
	yes	-	35.85 ± 5.07	
	-	-	14.80 ± 1.58	Guimarães et al., 2018 [30]
Bond strength (Mpa)	no	-	2.54 ± 1.26	Aguiar et al., 2019 [31]
	yes	-	4.13 ± 2.43	
Film thickness (μm) 150 N was applied	-	10 min	194 ± 89	Galarça et al., 2018 [29]
Exterior Volume (cm3)	-	-	0.0877 ± 0.0045	Guimarães et al., 2018 [30]
Volume of Open Pores (cm3)	-	-	0.0258 ± 0.0006	
Volume of Impervious Portion (cm3)	--	-	0.0619 ± 0.0044	
Apparent Porosity (Vop/V %)	-	-	29.45 ± 1.49	
Failure type- Adhesive (%)	no	-	56.25	Aguiar et al., 2019 [31]
Failure type- Adhesive (%)	yes	-	25	
Failure type- Cohesive (%)	no	-	37.5	
Failure type- Cohesive (%)	yes	-	25	
Failure type-Mixed (adhesive and cohesive) (%)	yes	-	6.25	
Failure type-Mixed (adhesive and cohesive) (%)	no	-	50	
interface of adaptation to the dentin wall (% of gaps)	no	-	28.58 (8.01–63.73) Median (min.-max.)	
interface of adaptation to the dentin wall (% of gaps)	yes	-	17.87 (0.0–43.26) Median (min.-max.)	
discoloration	yes	7 days	2.77 ± 1.12	
	no	7 days	1.95 ± 1.38	
	yes	15 days	2.70 ± 0.74	
	no	15 days	1.75 ± 1.46	
	yes	30 days	3.26 ± 1.47	
	no	30 days	1.44 ± 1.33	
	yes	180 days	2.60 ± 0.79	
	no	180 days	1.68 ± 0.67	
Evaluation of Microleakage values of leaked glucose (mM/L)	-	1 day	0.083 ± 0.005	Çırakoğlu et al., 2020 [33]
	-	10 days	3.644 ± 6.164	
	-	20 days	5.043 ± 3.663	
Radiopacity (mm/Al)	-	-	3.04 ± 0.16	Galarça et al., 2018 [29]
	-	-	4.50 ± 0.46	Guimarães et al., 2018 [30]

**Table 7:** Selected biological parameters of the MTA HP.

Biological Parameter	Additional Parameter	Results	Type of Research	Period Time	Tissue/Cell	Species	Number of Samples	Source
Inflammatory response	-	no activation of THP-1 monocytes not alter MMP-2 and MMP-9 protein expression	In vitro	48 h	monocytes and macrophages from THP-1 cells	Human	-	Barczak et al., 2021 [12]
	-	discrete	In vivo	4 weeks	skin and subcutaneous tissues	Wistar rats	25	Elreash et al., 2019 [9]
	-	moderate-few inflamation		30–90 days			8	Benetti et al., 2019 [21]
	-	no response		30 days			8	Benetti et al., 2018 [20]
	Hypertension Normo-tention	increase inflammation decrease inflammation		30 days			16 sponta-neously hyper-tensive (SHR) 16 normo-tensive (NT)	Cosme-Silva et al., 2019 [18]
Cytotoxicity	-	cell viability similar or higher than the negative control	In vitro	24 h	osteoblastic cells	Human	96 plates	Queiroz et al., 2021 [25]
	-	a significant increase in compare to control group (coulture complete medium without MTA HP)		5 days	dental pulp stem cells		21	El Reash et al. 2021 [28]
	-	no cytotoxic effect		72 h	dental pulp stem cells		10	Tomás-Catalá et al., 2017 [23]
	pH 5,2 dilutions 1:1 1:2 1:4	depends on the pH and degree of material dilution.		72 h	periodontal ligament stem cells		10	Collado-Gonzlez et al., 2016 [26]
		depends on the degree of material dilution.		72 h	bone primary osteoblasts		10	Ferreira et al. 2019 [6]
		depends on the degree of material dilution.		48 h	Fibroblast-like cells	Mouse	96 plates	Benetti et al., 2019 [21]
		discrete inflammation	In vivo	30 days	skin and subcutaneous tissues	Wistar rats	10	Ferreira et al. 2019 [6]
Apoptosis/necrosis	-	>94% of viable cells	In vitro	72 h	bone primary osteoblasts	Human	10	Ferreira et al. 2019 [6]
		96% of viable cells		72 h	periodontal ligament stem cells		10	Collado-Gonzlez et al., 2016 [26]
	-	discrete	In vivo	4 weeks	Skin and ubcutaneous tissues	Wistar rats	25	Elreash et al., 2019 [9]
Cell attachment on material	dilutions 1:1 1:2 1:4	good attachment to the material	In vitro	72 h	bone primary osteoblasts	Human	10	Ferreira et al. 2019 [6]
	-	Worse attachment than Neo MTA and Biodentine		72 h	dental pulp stem cells		10	Tomás-Catalá et al., 2017 [23]
	-	good attachment to the material		7 days	dental pulp stem cells		21	El Reash et al. 2021 [28]
	pH 5,2 dilutions 1:1 1:2 1:4	non depends on pH and degree of material dilution.		72 h	periodontal ligament stem cells		10	Collado-Gonzlez et al., 2016 [26]
Minera-lization		significant increases in the amounts of mineralizing nodules formed compare to control group	In vitro	7 days	dental pulp stem cells	Human	21	El Reash et al. 2021 [28]
		more mineralization noodles than control group		24 h	osteoblastic cells		96 plates	Queiroz et al., 2021 [25]
	Hypertension Normo-tension	decrease biomineralization increase biomineralization	In vivo	30 days	skin and subcutaneous tissues	Wistar rats	16 spontaneously hypertensive (SHR) 16 normotensive (NT)	El Reash et al. 2021 [28]
	*-*	100% biomineralization ability		30–90 days	skin and subcutaneous tissues		8	Benetti et al., 2019 [21]
		100% biomineralization ability		30 days	skin and subcutaneous tissues		8	Benetti et al. 2018 [20]
Antimicrobal effect	*E. faecalis*	positive activity	In vitro	48 h	osteoblastic cells	Human	96 plates	Queiroz et al., 2021 [25]
	*E. faecalis* *E. faecum* *S. aureus* *P. gingivalis* *C. albicans* *A. israelii* *P. anaerobius* *S. mutans*	positive activity positive activity positive activity positive activity positive activity negative activity negative activity negative activity		48 h	dental pulp stem cells		21	El Reash et al., 2019 [27]

**Table 8:** Summary of MTA HP articles.

Nr	Source	In Vitro/In Vivo	Physicochemical Properties	Biological Properities	Type of Research
1.	Silva et al., 2016 [5]	In vitro	Bond strength	-	Comparative Study
2.	Galarça et al., 2018 [29]	In vitro	Film thickness, flow, setting time, compressive strength	Cell viability	Research Article
3.	Guimarães et al., 2018 [30]	-	Radiopacity, calcium release, water sorption, solubility	-	Comparative Study
4.	Aguair et al., 2019 [31]	In vitro	Bond strength, marginal adaptation, tooth discoloration	-	Comparative Study
5.	Acris De Carvalho et al., 2021 [32]	-	Setting time, flow, dimensional change, solubility	-	Comparative Study
6.	Çırakoğlu et al., 2020 [33]	In vitro	Microleakage	-	Comparative Study
7.	Meraji et al., 2020 [34]	In vitro	Microhardness, microstructure	-	Comparative Study
8.	Metlerska et al., 2021 [35]	In vitro	Color Changes	-	Comparative Study
9.	Jiménez-Sánchez et al., 2019 [36]	-	Chemical composition, Hydration performance	-	Research Article
10.	Jiménez-Sánchez et al., 2020 [37]	In vitro	Hydration performance	Bioactive response	Comparative Study
11.	Jiménez-Sánchez et al., 2019 [38]	In vitro	Microstructural features	Bioactive response	Research Article
12.	Jiménez-Sánchez et al., 2019 [39]	In vitro	-	Mineralization	Research Article
13.	Barczak et al., 2021 [12]	In vitro	-	Inflammatory response	Research Article
14.	Tomás-Catalá et al., 2017 [23]	In vitro	Chemical composition	Cell viability, cell migration, cell morphology, cell attachment	Comparative Study
15.	Tomás-Catalá et al., 2018 [24]	In vitro	Chemical composition	Cell viability, cell migration, cell morphology, cell attachment	Comparative Study
16.	Queiroz et al., 2021 [25]	In vitro	Setting time, radiopacity, pH, solubility	Cytotoxicity, cell bioactivity, alkaline phosphatase activity, Alzarin red staining (ARS), real time PCR (qPCR), antibacterial activity	Comparative Study
17.	Collado-Gonzlez et al., 2016 [26]	In vitro	Chemical composition	Cell viability, aapoptosis, cell attachment	Comparative Study
18.	El Reash et al., 2019 [27]	In vitro	pH	Antimicrobial effect	Comparative Study
19.	El Reash et al., 2021 [28]	In vitro	-	Cytotoxicity, Cell attachment, mineralization	Comparative Study
20.	Benetti et al., 2019 [21]	In vitro/In vivo	-	Cytotoxicity, biocompatibility, biomineralization	Comparative Study
21.	Benetti et al., 2018 [20]	In vivo	-	Biocompatibility, biomineralization	Comparative Study
22.	Ferreira et al., 2019 [6]	In vivo	Setting time, flow, radiopacity, solubility	Cytotoxicity, Apoptosis, cell adhesion	Comparative Study
23.	El Reash et al., 2019 [9]	In vivo	-	Inflammatory response	Comparative Study
24.	Cosme-Silva et al., 2019 [18]	In vivo	-	Biocompatibility, biomineralization	Comparative Study
25.	Delfino et al., 2021 [19]	In vivo	-	Bioactivity, biocompatibility	Comparative Study
26.	Macedo et al., 2020 [22]	In vivo	-	Biomineralization	Research Article

## Data Availability

Data sharing is not applicable to this article.
